# Egalitarian Values and Sexual Behavior—The Role of Country Level Values in Shaping Individual Level Behaviors in Africa, South America and Asia

**DOI:** 10.1080/19317611.2021.1919952

**Published:** 2021-05-12

**Authors:** Anna Valverius, Linda Arnell, Mattias Strandh

**Affiliations:** a Department of Social Work, Umeå University, Umeå, Sweden; b School of Law, Psychology and Social work, Örebro Univeristy, Örebro, Sweden

**Keywords:** Egalitarian values, sexual behaviors, condom use, sexual activity, sexual well-being

## Abstract

Objective

The aim of this study is to investigate the relationship between country level egalitarian values (broadly speaking emancipatory values/structural gender equality) and sexual behavior among youth. *Methods:* Comparative individual level data on sexual activity and condom use were collected from the Global School-based Student Health Survey, resulting in a final sample of 23 countries, analyzed utilizing multilevel logistic regression analysis. *Results:* Egalitarian values were significantly associated with sexual activity and condom use. *Conclusions:* Egalitarian values have a relationship with adolescents’ sexual activity and condom use, and thus contribute to sexual health and well-being in Africa, South America and Asia.

## Introduction

Sexuality is an integral part of being human, and good sexual health and well-being are generally regarded as desirable (Casique, 2019; Harden, 2014; World Health Organization, 2002). Sexual well-being among youth is also associated with other aspects of their health, such as having a more positive body image and reducing psychological distress levels in both genders (Boislard et al., 2016). At the same time though, boys are generally allowed more sexual freedom, whereas sexual norms are often stricter for girls (Boislard et al., 2016; Marston & King, 2006). Unequal sexual norms such as differences in how, when and under which circumstances to have intercourse (for instance affecting casual intercourse, intercourse with many/few/one partner, etc.) are factors affecting sexual behaviors associated with, for instance, inconsistent contraceptive use (Leung & MacDonald, 2018). Egalitarian values could act as a counterweight to this, affecting for example the consistency of condom use, increasing the sense of personal responsibility, spreading positive sexual ideas and experiences thereof (Casique, 2019; Curtin et al., 2011; Leung & MacDonald, 2018). Egalitarian values are generelly based on one essential belief: that all humans are equal and should have equal rights and opportunities. The impact of egalitarian values on sexual behaviors has previously been studied at the “individual level.” Research has shown that equality is associated with increased communication and intimacy although it has been noted that this relationship is still somewhat under-investigated and unsubstantiated (Carlson & Soller, 2019). Individual level values, however, exist within contextual pre-conditions that can be measured at “the country level.” These contextual pre-conditions could for instance involve the gender equality in the country (that could be measured through several international indexes such as Gender Inequality index), the level of egalitarian values in the society and the level of human development of a country. Yet there is currently very little research focused on egalitarian values measured at a country level, and the potential effect the country level context has on youth sexual behavior and sexual health.

The aim of this study is to build on previous individual level research and investigate the role of egalitarian values at the country level in shaping youth individual level sexual activity and condom use. This is done using comparative survey data from the Global School-based Student Health Survey (GSHS) combining individual level data on sexual behavior and individual characteristics such as sex and experience of hunger from 23 non-Western low- and middle-income countries from Africa, South America and Asia. The individual level data is combined with country level indexes on egalitarian values. The overall gender equality in respective country is measured through the Gender Inequality Index (GII) and gender egalitarian values are measured through the Emancipatual Values Index (EVI) while the relative size of the country’s economy is measured through GDP/ppp. The inclusion of both GII and EVI to indicate country level egalitarian values was made in order to accommodate the potential importance of both structural egalitarian factors (such as women in government, chances to receive education for girls, measured by GII) and normative frameworks (such as a person’s autonomy, the ability to control everyday life decisions in relationships and marriage, measured by EVI). Previous research has shown that these can be quite separate as was the case in many post-communist countries (Haney, 2003). This means that it is possible that GII and EVI together are a better indicator of how egalitarian a society is, while it is also possible that they could be of differential importance.

The main contribution of this article is that it adds to the sparse literature on the role of contextual factors measured at the country level for individual level sexual behavior in general and for condom use in particular. The authors have found no previous comparative studies on contextual factors measured at the country level for condom use, a possible key factor for sexual well-being. An added benefit of the article is the significant addition it makes to our knowledge about sexual behavior in the low- and middle-income non-Western country contexts studied, as research investigating sexuality in areas such as South America, Asia and Africa is largely lacking (Boislard et al., 2016).

## Background

The World Health Organization (WHO) argues that sexual health includes mental, emotional, physical and social well-being, and is thus not only the *absence* of illness or sexual problems (World Health Organization, 2002). According to this definition, sexual well-being is a central aspect of sexual health. The term sexual well-being contains four focal aspects: sexual self-esteem, sexual self-efficacy, being able to express sensations concerning sexual feelings or experiences and experiencing sexuality in a healthy way, free of anxiety and/or pain (Casique, 2019; Harden, 2014). Sexual well-being also includes the right to say “no,” using contraceptives and being able to control how one’s sexuality is explored. This inclusive view of sexual health is related to what can be described as a sex positive framework. A sex positive framework considers sexuality as something that is natural and part of life, leading to increased health overall (Boislard et al., 2016; Harden, 2014; World Health Organization, 2002).

This broad definition of sexual health can encompass varied factors on all levels in a society, from country level to individual level. Given this potentially wide field of enquiry, there has been surprisingly little comparative empirical research on the relationship between equality measured at the country level and sexual behavior. There is, however, a significant body of descriptive literature comparing sexual behavior between multiple countries and studying how it changes over time. The findings from previous research indicate that major differences in sexual behavior between countries do exist and that over time there has been a general trend of later sexual debuting and increasingly positive attitudes to contraception and the use of contraception (Slaymaker et al., 2020; Wellings et al., 2006).

### Between country variations

Empirical studies looking at factors explaining between country variations in sexual behavior are very rare. The only study found by the present authors that explicitly compares several countries was performed by Baumeister and Mendoza (2011). Using an online survey conducted by the condom maker Durex with over 300,000 adult respondents (convenience selection), they compared data from the global gender gap report at the country level with reported sexual activity from 37 countries, finding that increased levels of sexual activity correlate with more egalitarian societies (Baumeister & Mendoza, 2011). Given the probably skewed sample in this study of individuals going online to find information about sex (individuals that are online as well as interested in sexual issues), there is a need to replicate this work using more randomized selections. One additional study was found comparing two countries, Bolivia and Ecuador, by De Meyer et al. (2014). Using a stratified selection of 5,913 adolescents aged 14–18 years in 20 secondary schools in Cochabamba (Bolivia) and six secondary schools in Cuenca (Ecuador), they conclude that country level gender equality is an important factor for contraceptive use. Additionally, gender equality also correlates with youth communicating more easily about their sexual activity, thus increasing sexual health overall. However, it is not a significant factor for increased or decreased sexual activity (De Meyer et al., 2014). Furthermore, neither of these studies takes the clustered data into consideration, and different data levels (country level variables and individual behavior variables) are not analytically dealt with through a multilevel analysis.

From a sex positive framework perspective, sexual activity, as studied by Baumeister and Mendoza (2011) and De Meyer et al. (2014), is an important indicator of sexual health. However, Baumeister and Mendoza (2011) do not measure several important aspects of sexual health, such as control and consent as well as safe sex, and in the De Meyer et al. (2014) study the clustered data are not treated as such. Consistent condom use has been associated with greater self-esteem and positive behaviors during sexual situations, such as taking responsibility and taking care of oneself (Casique, 2019). Overall, consistent condom use is understood to have a direct impact on sexual well-being as a positive behavior. Studies show that positive sexual experiences are associated with more consistent condom use (Casique, 2019; Curtin et al., 2011; Leung & MacDonald, 2018). Due to the limitations described above, we argue the need for additional research that takes different data levels into consideration. Particularly, there is a need for research that combines sexual activity with other aspects of sexual health (such as condom use) as an indicator of safe and healthy sex in order to investigate the role of contextual factors measured at a country level for individual’s sexual health. Context at the country level could affect how youth act and view themselves (self-efficacy, self-worth etc.) and thereby their sexual well-being.

### Social institutions and societal structures

Comparative research traditions indicate that the contextual level is important for individual attitudinal structures and behavioral constraints. New institutional theory emphasizes the role of social norms, rituals and roles for individual outcomes: a social institution is a system of patterns describing accepted behavior and actions which an individual can use to satisfy a societal need (Eriksson-Zetterquist, 2012; Vestheim & Kangas, 2010). This system of patterns describing accepted behavior and actions needs to be recurring over time to be described as a social institution (although the length of time needed to achieve that status is unclear). This framework, then, could be used to better understand sexual behaviors. The relevant contextual structures in a country or society could potentially indicate accepted behaviors and actions for an individual, for instance if an individual is entitled to vote or go on parental leave (two historically gendered actions). In contrast to the lack of research on country level factors and the differences and similarities between different countries and sexual behaviors, the current body of research concerning individual behaviors affected by structural contextual factors is rather large.

Earlier findings indicate that contextual factors related to condom availability and sex education affect sexual behaviors and consistent condom use. Firstly and obviously, condoms have to be available. Youth who can’t access condoms won’t use condoms (Jellema et al., 2013; Marston & King, 2006; Ryan et al., 2007). The ready availability of condoms for young people could also be of importance to establish long-lasting habits. It has been found that condom used during first sexual intercourse is associated with increased likelihood of consistent condom use for both women and men (Casique, 2019; Havaei et al., 2019). The second important factor for young people’s sexual behavior, measured on an individual level, is sexual health education (SHE) (Bedree et al., 2020; Foshay & O’Sullivan, 2020; Sun et al., 2018). Some research also argues that actual knowledge is not all that matters—perceived knowledge also affects whether contraceptives are used (Ryan et al., 2007). In accordance, if young people—especially but not exclusively boys—believe that they have extensive and in-depth knowledge about contraception then the chance of using contraceptives increases (Ryan et al., 2007). The difference in perceived knowledge may be partially explained by the social importance for men to achieve penetrative sex and thus appear as experienced (Marston & King, 2006).

### The role of equality and egalitarian values at the individual level

Even though it is currently unclear if egalitarian values measured at the country level is related to the frequency of sexual activity and condom use for the individual, egalitarian values measured at the individual level could play an important role. The need for a contextual perspective to understand sexual behavior more fully has previously been raised by Carlson and Soller (2019), who discussed the problems of assessing the importance of gender conventions for sexual scripts; in particular, they questioned the association of sexual frequency with gendered ideology due to a lack of research. According to Carlson and Soller (2019), there are two lines of reasoning with no clear conclusion. On the one hand, conventional behaviors trigger sexual scripts and seem to elicit arousal, emphasizing the mens’ role to initiate intercourse and stay in control. Since sex is a way of “doing gender,” following scripts could lead to increased sexual activity, and this would increase sexual activity for those who share less egalitarian values. On the other hand, open communication and women who view themselves as active sexual partners could increase the frequency of sexual activity for those who espouse more egalitarian values. The results from Carlson and Soller’s (2019) own individual level research show that egalitarian attitudes toward domestic and paid labor and the attitudes of men toward equality together have a negative association with the frequency of sexual activity and womens’ self-efficacy, while mens’ sexual control has a positive association with the frequency of sexual activity. In other words: there are good arguments for both traditional normative behavior, as well as increased female sexual self-efficacy, increase sexual activity at the individual level. Which of these mechanisms turn out to be the stronger factor in relation to country level values remain unknown and is an empirical question.

The presented role of the normative framework above can also be seen in findings on the relationship between traditional values and safe sexual behaviors. Traditional values stress deeply rooted family values (such as the nuclear family with two heterosexual parents), the significance of religion, parent-child ties and respect for authority (Inglehart et al., 2014). These values, in general, conflict with egalitarian values since liberation and viewing all humans as equal typically includes questioning tradition, religion and authority. A common general concern for girls and women is the belief that it is inappropriate for them to suggest the use of condoms, a fear that their partner will appreciate them less if the they propose condom use (Marston & King, 2006; Vasilenko et al., 2015). This can be referred to as an “embarrassment barrier” for women, in other words a barrier inhibiting the carrying, accessing and purchasing of condoms (Bell, 2009; Jellema et al., 2013; Leung & MacDonald, 2018; Marston & King, 2006). This effect could be related to what sometimes is referred to as the “double standard.”

The double standard means that contraceptive-prepared women and men are viewed differently: women are negatively perceived as promiscuous whereas men are not (Berrocal et al., 2019; Marston & King, 2006; Pearson, 2018; Vasilenko et al., 2015). Something that throughout history has caused women to be seen negatively is public knowledge of their being sexually active (Leung & MacDonald, 2018); probably because of this some women pretend that they know nothing about contraceptives in order to make sure that their reputation does not become damaged (Marston & King, 2006). In terms of behaviors, traditional gender roles and similar attitudes can become an obstacle to sexual health, for example by diminishing women’s sexual autonomy and thereby reducing satisfaction, discouraging contraceptive use and increasing the risk of sexual violence (Casique, 2019; Grose et al., 2014; Pearson, 2018; Shannon et al., 2012). Finally, less ability to access and utilize sexual-risk knowledge, advocate for their own sexuality and condom self-efficacy have been related to women who endorse traditional values and lower rates of condom use are reported by these women in comparison to men (Leung & MacDonald, 2018). Furthermore, hostile attitudes toward women in markedly traditional societies make men more likely to resist condom use in heterosexual intercourse (Curtin et al., 2011; Grose et al., 2014; Leung & MacDonald, 2018).

## Aim of the study

The aim of this study is to build on previous individual level research and investigate the role of egalitarian values at the country level in shaping youth individual level sexual activity and condom use. The research questions are:

1. Is sexual activity among adolescents related to egalitarian values at the country level?
2. Is the relationship between egalitarian values at the country level and sexual activity larger, smaller or the same for girls’ and boys’?
3. Are sexually active adolescents in egalitarian societies using condoms more often than their peers in less egalitarian societies?
4. Is the relationship between egalitarian values at the country level and condom use larger, smaller or the same for girls’ and boys’?

## Data, variables and methods

### Data

The aim of this article is to investigate the role of country level factors (specifically egalitarian values) on the individual sexual behavior of young people. This requires access to individual level data containing comparable information on young peoples’ sexual behaviors from multiple country contexts. The GSHS offers a unique data source providing a harmonized comparative individual level data set on students’ health behaviors. The GSHS was initiated by the WHO and the Centers for Disease Control and Prevention (CDC) in collaboration with UNAIDS, UNESCO and UNICEF (Centers for Disease Control and Prevention, 2013). GSHSs are conducted in approximately 110 countries, with fact sheets and reports available for 103 of them, the majority being low- or middle-income areas. The GSHS questionnaire is a self-reported survey conducted primarily among students between the ages of 13–17 (some students being a couple years older or younger). The samples are selected on a national or regional basis, or sometimes both combined (Assarsson et al., 2018; Centers for Disease Control and Prevention, 2013).

To measure contextual country level egalitarian values data from the World Values Survey (WVS) and the Gender Inequality Index (GII) created by the United Nations Development Program (UNDP) were chosen. The WVS is a comparative survey of values with a robust methodology; it is easily available online and covers many of the countries that are present in the GSHS. The WVS began as a part of the European Values Study in 1981 and soon became a widely used cross-national survey covering almost 100 societies (Inglehart et al., 2014). The WVS focuses on and explores peoples’ beliefs and values, changes to them over time and their impact on political and social development. All data available for public use are approved by the WVS board. The data need to be randomly sampled with a minimum of 1200 complete interviews and be representative for all residents between the ages of 18 and 85 for each country (Inglehart et al., 2014).

Country level information from the WVS and UNDP was matched to the individual level GSHS data. In most cases, the information was not available for the actual GSHS interview year in each country, but the information was still matched as closely as possible. Some countries were excluded because the time difference in data collection between the GSHS and WVS/UNDP was judged to be too big. The final sample only contains countries where the data from the GSHS, WVS and UNDP were collected within a time span of 6 years. During the matching process, it was possible to match some countries from the GSHS, UNDP and WVS twice. During data cleaning it could be observed that both individual level and macro-level measures changed substantially within the same country in between interview waves of GSHSs. Since the aim of this study was to investigate the effect of egalitarian values on sexual behaviors, these were, in the analyses, treated as separate observations and are referred to as Matching I and Matching II. Sensitivity tests were made to evaluate this choice.

The final sample includes 17 different countries, with six being included twice for a total of 23 country contexts. The total number of individuals analyzed is 286,560. Descriptive statistics for the dependent variables and the macro variables of all countries can be seen in Appendix Table S1 and Table S2.

**Table 1.** Multilevel logistic regression: the effect of country level egalitarian values on sexual activity.

	BOTH GENDERS (A)					BOYS (B)					GIRLS (C)				
MODEL	1	2	3	4	5	1	2	3	4	5	1	2	3	4	5
LEVEL 1															
AGE															
≤12 YEARS		0b	0b	0b	0b		0b	0b	0b	0b		0b	0b	0b	0b
13 YEARS		0.26	0.27	0.26	0.27		0.31	0.32	0.31	0.32		1.19	1.19	1.19	1.19
14 YEARS		0.70***	0.70***	0.70***	0.71***		0.73***	0.74***	0.73***	0.74***		0.67***	0.67***	0.67***	0.67***
15 YEARS		1.21***	1.21***	1.21***	1.21***		1.22***	1.22***	1.22***	1.22***		1.19***	1.20***	1.19***	1.20***
≥16 YEARS		1.77***	1.77**	1.77**	1.77**		1.74***	1.73***	1.74***	1.73***		1.81***	1.81***	1.81***	1.81***
GIRLS		−0.72***	−0.71***	−0.72***	−0.71***		0b	0b	0b	0b		0b	0b	0b	0b
BOYS		0b	0b	0b	0b		0b	0b	0b	0b		0b	0b	0b	0b
HUNGER		0.12***	0.12***	0.12***	0.12***		0.11***	0.11***	0.11***	0.11***		0.13***	0.13***	0.13***	0.13***
LEVEL 2															
GDP/ppp			−0.03	0.00	−0.04			−0.03	0.01	−0.04			0.05	0.01	−0.05*
GII			−0.01		−0.01			−0.01		0.02			−0.02		0.00
EVI				0.07*	0.07**				0.07*	0.08**				0.07*	0.07**
*N* COUNTRIES	23	22	21	22	21	23	22	21	22	21	23	22	21	22	21
INTERCEPT	−1.62***	−2.47***	−1.86	−5.22***	−6.00***	−1,22***	−2.41***	−2.05	−5.27***	−6.68***	−2.14***	−3.31***	−2.29***	−6.30***	−6.52***
AKAIKE	1,134,049	1,132,142	1,111,893	1,132,108	1,111,790	509,763	508,569	499,882	508,579	499,812	629,730	626,395	614,244	626,357	614,167

*p ≤ 0.05; **p ≤ 0.01; ***p ≤ 0.001.

Metric used to interpret results: log odds coefficients.

0b: reference category.

**Table 2.** Multilevel logistic regression: the effect of country level egalitarian values on condom use.

	BOTH GENDERS (A)					BOYS (B)					GIRLS (C)				
MODEL	1	2	3	4	5	1	2	3	4	5	1	2	3	4	5
LEVEL 1															
AGE															
≤12 YEARS		0b	0b	0b	0b		0b	0b	0b	0b		0b	0b	0b	0b
13 YEARS		−0.15	−0.15	−0.15	−0.15		−0.17	−0.16	−0.17	−0.16		−0.06	−0.10	−0.06	−0.10
14 YEARS		−0.01	0.00	−0.01	0.00		−0.01	0.01	−0.01	0.01		0.01	−0.02	0.01	−0.02
15 YEARS		0.00	−0.01	−0.01	−0.01		0.11	0.12	0.11	0.12		−0.15	−0.18	−0.15	−0.18
≥16 YEARS		−0.08	−0.08	−0.08	−0.08		0.02	0.03	0.02	0.03		−0.20	−0.24	−0.21	−0.24
GIRLS		−0.25***	−0.26***	−0.25***	−0.26***		0b	0b	0b	0b		0b	0b	0b	0b
BOYS		0b	0b	0b	0b		0b	0b	0b	0b		0b	0b	0b	0b
HUNGER		−0.09**	−0.09**	−0.09**	−0.09**		−0.10**	−0.10**	−0.10**	−0.10**		−0.08**	−0.08*	−0.08*	−0.08*
LEVEL 2															
GDP/ppp			0.02	0.02	0.02			0.03	0.01	0.03			0.01	0.02	0.00
GII			0.01		−0.01			−0.01		0.00			0.01		0.02
EVI				0.04	0.04*				0.04*	0.04*				0.03	0.04*
*N* COUNTRIES	23	22	21	22	21	23	22	21	22	21	23	22	21	22	21
INTERCEPT	0.33*	0.66**	0.81	−0.91	−0.67	0.40**	0.60**	0.98	−1.09	−1.40	0.02	0.52	0.21	−0.60	−2.22
AKAIKE	174295	164867	162726	164895	162735	103982	99288	97584	99317	97593	68080	65764	65348	65792	65356

*p ≤ 0.05; **p ≤ 0.01; ***p ≤ 0.001.

Metric used to interpret results: log odds coefficients.

0b: reference category.

### Individual level variables

All the individual level variables were collected from the GSHS. Two main dependent variables measuring sexual behaviors were used. To measure sexual activity, the question “Have you ever had sexual intercourse?” was selected with the possible answers of yes or no. To measure condom use, the question “The last time you had sexual intercourse, did you or your partner use a condom?” was selected with the possible answers yes, no or no intercourse. There were small variations in the questions since the word condom was translated into multiple languages, and surveys utilizing verified synonyms for the word condom were included. As an aside, it is worth remarking that it would have been good if multiple contraceptive practices could have been studied. A few of the survey waves had multiple questions focusing on different contraceptive methods, but the questions were asked in several different ways and were deemed to be not comparable; to ensure the quality of the analyses and keep a high consistency these were excluded.

To control for compositional differences between countries three additional individual level confounders were used. The timing of sexual intercourse is a heavily researched factor, and there also seem to be clear differences between women and men both in terms of activity and use of contraceptives (Boislard et al., 2016; Leung & MacDonald, 2018). Therefore, age and sex were chosen. A question measuring hunger due to lack of food at home was also included since it could indicate different material assets, which have been shown to affect the desire for democracy and freedom of choice (Inglehart et al., 2014). Age was measured with a categorical scale: 11 years or younger, 12, 13, 14, 15 and finally 16 years or older. Since the two youngest categories were small in comparison to the other categories, they were combined into the category 12 years or younger. Sex was measured with the question “What is your sex?” and the possible answers woman or man. Hunger due to lack of food at home was measured with the question “During the past 30 days, how often did you go hungry because there was not enough food in your home?” and the possible answers were sorted as a categorical scale ranging from “never” to “always” with five alternatives. During the cleaning of the data this scale was changed from a categorical to numeric scale. This was tested during the sensitivity tests and it did not change the overall results but made interpretation of the results clearer.

### Country level variables

Since egalitarianism is based on the fundamental belief in equal rights and opportunities for all, emancipation is logically the basis for an egalitarian society. There is thus a need to study both structural gender inequality and emancipation—after all, a high level of equality measured by one measurement is not a guarantee for every part of a society being equal.

To measure structural gender inequality, we used the GII constructed by the UNDP. The GII measures loss in potential human development between women and men at the country level (Gunkelman, 2002); it covers the following factors: maternal mortality rate, adolescent birth rate, share of parliamentary seats held by each sex, population with at least some secondary education (SE) and labor force participation rate (Gunkelman, 2002). The index ranges from 0 to 1: if women and men fare equally in those elements the GII score is 1 and thus GII 0 indicates complete inequality with one gender doing poorly in all measured elements. To increase the readability of the index all GII scores were multiplied with 100, creating a scale 1–100 where observed values range from 30 to 62. Other indexes measuring structural gender inequality were also considered, namely the Inequality-adjusted Human Development Index (IHDI), the Human Development Index (HDI) and Gender Equality Measurement (GEM). After due consideration, the IHDI and HDI were disqualified since important measurements of political dimensions were lacking; they also have strong similarities with the GII (Klugman & Choi, 2011). The GEM was replaced by the GII in 2010; the latter is regarded as more accurate and correct in relation to some of the drawbacks associated with the GEM (Klugman, 2010).

To measure the values important for an egalitarian society the Emancipative Values Index (EVI) from the WVS measured at the country level was used. The EVI measures perceived freedom of choice, equal opportunities and values related to lifestyle, gender equality and personal autonomy (Inglehart et al., 2014). Specifically, it includes views on parenting methods and the freedom the child should have, gender equality values in relation to the labor market and leadership, the acceptance of homosexuality, acceptance of abortion, acceptance of divorce and the importance of freedom of speech. The index range is from 0 to a maximum 1.0; a low score indicates weaker values, a high score indicates stronger values (Inglehart et al., 2014; Welzel, 2013). A high score on EVI stands in opposition to conservative and/or traditional societies. This index was also multiplied by 100 for the same reasons as the GII.

Lastly, it was deemed necessary to control for country level material conditions since this is not included in either the GII or the EVI. Here GDP/ppp as calculated by the World Bank was used. GDP/ppp measures gross domestic product based on purchasing power parities. This measurement is usually used to measure standards of living between countries (World Bank, 2019). To simplify the understanding of GDP/ppp all scores were divided by 1000.

## Procedure for analysis

The data used for this analysis was pooled from many countries and different sources. Because of this it has a nested structure with individuals (level 1) nested in different countries (level 2). Consequently, each observation or individual could possibly be affected by the nested structure, rendering ordinary least square regression analysis sub-optimal (Högberg et al., 2019). Since the data are nested, individual observations are not independent of each other. Multilevel regression analysis allows for observations of how level 1 variables and level 2 variables affect the dependent variable. These steps make analysis of how clusters are affected possible since level 1 independent variables and the dependent variable can be conditional on the value of level 2 variables (in this case country).

Within the multilevel framework logistic regression was used, as the dependent variable was binary. Several metrics can be used to interpret results from logistic regression models; for this article log odds coefficients (b) were chosen and used in combination with a calculated percentage to simplify the understanding of the coefficients. The predicted log odds should be interpreted as the log of the odds between the observed category and the reference category within the dependent variable in relation to the studied event (individual in x country had sex/used condom) when all other individual predictors are set to zero and the random effect is held consistently at zero (Heck et al., 2012).

Level 1 variables used for the analyses were sex, age and level of hunger. Level 2 variables used for the analyses were GDP/ppp, GII and EVI. To observe the best-fit model and compare the models, log likelihood was utilized combined with the Akaike information criterion (AIC). The AIC provides a penalty based on the complexity of the model, multiplying the number of parameters (p) by two and adding this result to the error term also called deviance statistic (Heck et al., 2014). If the sample size is above 100 individuals the AIC has been noted to work well in selecting the best-fit model (Heck et al., 2014). All calculations were done using SPSS 2.6 GENLIN MIXED. When selecting the test of fixed effects and coefficients the setting “use robust estimation to handle violations of model assumptions” was used. The robust standard errors model is generally more conservative regarding possible variations from normality (Heck et al., 2012). Since underestimating the standard errors could lead to more findings of significance, the robust model uses more caution when calculating the standard errors (Heck et al., 2012). Sensitivity tests were done to evaluate this choice.

## Results

### Structure of tables

Table 1 presents the multilevel logistic regression models for research questions 1 and 2 where sexual activity is investigated, and Table 2 presents the multilevel logistic regressions for research questions 3 and 4 where condom use is investigated. Both Tables share the same structure. For each research question there are five models. The first model is empty in order to observe if there is indications of a contextual phenomenon (Merlo et al., 2006)—in other words, whether there is a significant difference between countries based on the dependent variable. Thereafter the second model includes all level 1 variables. The second model is utilized in order to observe the differences between countries explained by the composition of individuals within each country (Merlo et al., 2006). The third, fourth and fifth models include level 2 variables where GDP/ppp (which is treated as a confounder) is constant and GII and EVI are calculated one by one and then together. The level 2 variables were utilized one by one and then together to handle potential multicollinearity. In each Table the result is presented in three parts. Tables 1(A) and 2(A) present the results for both girls and boys together, Tables 1(B) and 2(B) only boys and Tables 1(C) and 2(C) only girls.

### Sexual activity

Starting with sexual activity in Table 1(A), model 1 was significant, thus a difference between the countries due to contextual factors was observed. Turning to model 2, where the individual level variables are entered, the individual level factors affecting sexual activity were age, level of hunger and sex. Compared with the reference group (12 years or younger) all age groups were significant except 13 years old. The odds of having intercourse at age 14 years increase by approximately 161% (*b* = 0.70–0.71), 15 years by approximately 234% (*b* = 1.21) and 16 years or older by approximately 485% (*b* = 1.77). To analyze sex, girls were compared with boys. Girls had approximately 51.1% (*b* = −0.72) decreased log odds of having intercourse. For hunger, a 1-point increase on the scale increased the log odds of having intercourse by approximately 12.7% (*b* = 0.12). Turning to models 3–5, when the level 2 variables were introduced many of the individual level variables did not change.

Continuing with models 3–5, and looking at the second level variables, neither GDP/ppp nor GII was significantly associated with sexual activity. EVI, on the other hand, was found to have a significant positive association with sexual activity in both models 4 and 5, and increased the odds of sexual activity by approximately 7.4% for each step of the scale (model 4 *b* = 0.07: model 5 *b* = 0.08). Analyzing boys and girls separately in Tables 1(B) and 1(C) there was almost no difference in the effect of the second level variables.

### Condom use

In the next table, Table 2(A), the probability of using condoms among those youths who had sexual intercourse is investigated. Beginning with model 1 the coefficient was significant thus a difference between the countries due to contextual factors was observed. Turning to model 2, where the individual level variables are entered, the factors significantly affecting condom use were sex and level of hunger. Girls had decreased odds of using condoms, with approximately 21.8% (*b* = −0.25). For hunger, a 1-point increase on the scale decreased the odds of using condoms by approximately 8.7% (*b* = −0.09). The effects of the individual level variables did not change when the level 2 variables were introduced in models 3–5.

Continuing with models 3–5, and looking at the second level variables, neither GDP/ppp nor GII was significantly associated with condom use. EVI, on the other hand, was found to have a significant association with condom use in model 5 and increased the odds to use condoms by approximately 4.3% for each step of the scale (*b* = 0.04). Analyzing boys and girls separately in Tables 2(B) and 2(C) there was almost no difference in the effect of the second level variables. The most notable change was that EVI was significant in models 4 and 5 in Table 2(B), indicating a more consistent relationship between EVI and boys’ use of condoms.

### Sensitivity tests

Several sensitivity tests were performed to ensure the quality of the analyses and the robustness of the results. The sensitivity analyses were made utilizing the same multilevel logistic regression approach but excluding different countries to determine if this impacted the results. These analyses were made to make sure that the results are not driven by individual country observations. First, an analysis was made with all countries and compared to an analysis using each country once, including the most recent country context (Matching II). No significant change was observed when analyzing sexual activity; some changes were noticed when analyzing condom use. In addition to EVI being significant in all models, GII became significant. When looking more closely at the variance between Matching I and Matching II the difference in the variable measuring condom use is smaller than the variable measuring sexual activity. Thus, it is up for debate whether the results of the models with both Matchings or the models with Matching II are better. To avoid type II errors (false positives), we decided to stick to the model with both Matching I and Matching II to evaluate the results.

Secondly, the impact of the outliers was tested by excluding them. No significant change was observed when analyzing sexual activity, but some changes were noticed when analyzing condom use. Either GII or EVI were significant in all models: in other words, one of the variables measuring egalitarian values was significant in all models, but neither GII nor EVI were consistently significant.

Thirdly, all regressions were made assuming model assumptions to be correct and comparing the results with the robust model’s assumptions. Some differences were observed. The significant associations were similar but stronger, assuming model assumptions to be correct in almost all cases except boys’ condom use. EVI was not significant in the models analyzing boys’ condom use but was significant when analyzing girls’ condom use. This could imply a slight risk of underestimating the associations using the robust model assumptions in all cases except boys’ condom use. However, in this case, the robust specifications were deemed to be preferable to avoid type II errors, the risk of which is a substantially larger problem than a slight underestimation of effects. Therefore, the robust model was used for the presented main analysis.

## Discussion

This article has investigated whether country level egalitarian values measured as emancipatory values (EVI) and structural gender inequality measured through the Gender Inequality Index (GII) affect sexual behaviors in terms of sexual activity and condom use. Four research questions were raised. The first two focused on whether sexual activity among adolescents was associated with egalitarian values at the country level and furthermore if this effect was gendered. The findings indicate that country level egalitarian values measured as emancipatory values increase the odds of having had sexual intercourse and that the effect is not gendered. No effect was found for structural gender equality.

The third and fourth research questions focused on whether sexually active adolescents in countries with prevailing egalitarian values used condoms more often than adolescents in countries with less egalitarian values, and once again if the effect was gendered. The findings are here a little less conclusive but do point toward an effect of egalitarian values on condom use. Both models used to analyze EVI separately and together with GII were significant for boys, but only the model analyzing EVI and GII separately was significant for girls and both genders analyzed together. In the sensitivity tests one of EVI or GII was significant in each model. When the most recent match of every country (17 country contexts) was analyzed both EVI and GII were however significant.

These results are in line with previous research where several studies (Marston & King, 2006; UNESCO, 2017; Vasilenko et al., 2015) illustrate how gender inequalities in a society could limit the access to and use of contraceptives, and gender equality increases sexual activity (Baumeister & Mendoza, 2011). It is possible that the lower rates of girls carrying, using and suggesting the use of condoms (Leung & MacDonald, 2018; Marston & King, 2006) could partly be explained by lack of equality and an absence of egalitarian values. It has been noted by multiple studies (Berrocal et al., 2019; Leung & MacDonald, 2018; Marston & King, 2006; Pearson, 2018; UNESCO, 2017; Vasilenko et al., 2015) that signs of girls’ sexual activity, carrying or use of contraceptives, could lead to stigma and/or embarrassment. UNESCO reinforce the importance of gender equality and discussion of power within relationships as crucial components of effective comprehensive sexuality education (CSE) (UNESCO, 2017). Furthermore, including gender in CSE renders it up to five times more effective in terms of its ability to significantly decrease sexually transmitted diseases and pregnancy (Haberland, 2015).

Another important contribution of this paper is that it emphasizes the need to analyze sexual behavior more fully by taking context into consideration. Egalitarian values are a part of a country’s social institutions, and it is well established that social institutions affect individual values and behavior (Eriksson-Zetterquist, 2012; Vestheim & Kangas, 2010). This reflection should, however, not be taken as an indication that change is impossible. Institutional factors evolve and change, and change is also possible under institutional constraints. One such example is education. Education has the potential to increase EVI, and previous research from other fields shows that it also has the ability to do this even when other social institutions do not favor EVI (Welzel, 2012). Indeed, it would be important to investigate this further, as well as looking at how changing values affect an individual’s actual behavior in social contexts where other social institutions do not favor egalitarian values.

### Limitations

This study had some limitations that need to be acknowledged and considered. All the data used was pooled from many sources. This means that the sampling process, wording and structure of the survey were definite. This created certain challenges during the cleaning of the data, and some countries were disqualified due to difficulties interpreting what the variables actually measured. Furthermore, the choice of which sex the participants identifies with was limited to girl or boy, assuming heteronormativity, thereby excluding youth identifying as queer or non-binary. It is an important limitation. Condom use is related to sexual health, even though some of the respondents might have been using additional forms of contraceptives and some of the youth who do not use condoms might use other forms of contraception. Much of the background work was done with this limitation in mind. The definite sampling process chosen by the GSHS, WVS and UNDP also limited which countries could be included within the timeframe of this study. It proved to be a challenge to find enough countries that could be matched (GSHS, WVS and UNDP) in a satisfactory way. The analyses would probably be more robust if more countries could have been matched. The countries selected for this study were the countries for which data was readily available—a random selection was not possible. This leads to one important point to note: this result is not representative for all countries. It is representative for the country contexts studied. Both GII and EVI scores varied in a satisfactory way, and the representation of countries in South America, Africa and Asia was robust and represents a significantly under-represented context in the generally Western-centric research that dominates the field. This article provides evidence that expands the contextual reach of previous research.

One additional limitation is important to have in mind: the GSHS is a school-based survey. Consequently, the results presented in this article are valid for adolescents going to school, not all adolescents. In some countries where fewer adolescents go to school, this fact may have significant implications. Since critical thinking, using logic and asking questions generally are part of most education systems, education has been shown to have the potential of increasing EVI (Welzel, 2012). It is possible that the associations presented in this article would be stronger if adolescents not attending a school were included.

On the other hand, the body of data used in this article is widely used in comparative research on youth values and behavior. Both the WVS and GSHS are transparently constructed as well as publicly available and based on robust methodology (Centers for Disease Control and Prevention, 2013; Inglehart et al., 2014). The other indexes (GII, GDP/ppp) used in this article are also widely used by others and are generally regarded as accurate measurements (Klugman, 2010). An additional strength is the extensive sensitivity testing, investigating the complexity of the data and ensuring the quality and robustness of the analysis. Thus, this study contributes to our understanding of the effects which egalitarian values have on sexual behaviors, the importance of context and our comprehension of egalitarian values outside of the Western world.

## Conclusion

Our conclusion is that egalitarian values have a relationship with both adolescents’ sexual activity and condom use. Results indicate that the effect of egalitarian values on sexual activity is not gendered; it is less clear whether the effect of egalitarian values on condom use is gendered or not and further research is needed to evaluate this particular matter. Furthermore, EVI measure aspects closely related to everyday life, whereas GII measure societal structures, not as explicitly present in a person’s everyday life. This could be of importance and a potential explanation for GII and EVI showing different results. Future research should analyze freedom of choice and forces restricting personal autonomy (conservatism, religion, etc.) in relation to sexual health for youth. These findings raise some interesting questions on the relationship between country level values and sexual behaviors; there is here a need for analysis of issues such as consent and sexual violence, which might need a qualitative comparative approach. 

## Supplementary Material

Supplemental Material

Supplemental Material

## Data Availability

Inglehart, R., C. Haerpfer, A. Moreno, C. Welzel, K. Kizilova, J. Diez-Medrano, M. Lagos, P. Norris, E. Ponarin & B. Puranen et al. (eds.). 2014. World Values Survey: Round Six – Country-Pooled Datafile Version. Madrid: JD Systems Institute. https://www.worldvaluessurvey.org/WVSDocumentationWV6.jsp. Inglehart, R., C. Haerpfer, A. Moreno, C. Welzel, K. Kizilova, J. Diez-Medrano, M. Lagos, P. Norris, E. Ponarin & B. Puranen et al. (eds.). 2014. World Values Survey: All Rounds – Country-Pooled Datafile Version. Madrid: JD Systems Institute. https://www.worldvaluessurvey.org/WVSDocumentationWVL.jsp. https://www.who.int/ncds/surveillance/gshs/datasets/en/
