# Supplementary material for: Egalitarian Values and Sexual Behavior—The Role of Country Level Values in Shaping Individual Level Behaviors in Africa, South America and Asia
Source: Int J Sex Health. 2021 May 12;33(3):396–409. doi: 10.1080/19317611.2021.1919952 (PMC10929577; doi:10.1080/19317611.2021.1919952)
Supplement: Supplemental Material [file WIJS_A_1919952_SM7270.docx]

| Table S2 Individual level sexual behaviors | | | |
| --- | --- | --- | --- |
| Ever had intercourse | | **Used condom last intercourse** | |
| Country | **Yes (%)** | **Country** | **Yes (%)** |
| Argentina II (n=52676 ) | 43.4 | Uruguay II (n=1978) | 81.6 |
| Zambia (n=1482) | 43 | North Macedonia (n=486) | 78.6 |
| Uruguay II (n=6768) | 33.7 | Argentina I (n=904) | 77.7 |
| Argentina I (n=3588) | 32.7 | Argentina II (n=19102) | 75.6 |
| Chile II (n=3932) | 32.3 | Bangladesh (n=39974) | 75.3 |
| Trinidad & Tobago II (n=5118) | 29.7 | Peru (n=1030) | 64.9 |
| Uganda (n=4230) | 28.6 | Uganda (n=1168) | 63.4 |
| Bolivia (n=146498) | 26.2 | Guatemala II (n=150534) | 61.7 |
| Trinidad & Tobago I (n=5244) | 24.7 | Thailand II (n=1042) | 60.3 |
| Guatemala II (n=150534) | 20.4 | Thailand I (n=280) | 59.3 |
| Peru (n=5638) | 20.1 | Guatemala I (n=1146) | 58.3 |
| Thailand II (n=10776) | 16.4 | Bolivia (n=146498) | 58.2 |
| Venezuela (n=6952) | 15.9 | Trinidad & Tobago I (n=1246) | 58 |
| Guatemala I (n=10538) | 15.3 | Trinidad & Tobago II (n=1110) | 57.5 |
| Chile I (n=14100) | 14.7 | Ghana (n=1286) | 54.9 |
| Zimbabwe (n=7474) | 14.2 | Venezuela (n=1094) | 54.7 |
| Ghana (n=9892) | 13.7 | Chile II (n=1020) | 53.1 |
| North Macedonia (n=3916) | 12.6 | Zambia (n=582) | 52.2 |
| Bangladesh (n=39974) | 8.2 | Chile I (n=2040) | 46.7 |
| Malaysia (n=47290) | 8 | Zimbabwe (n=1022) | 45.8 |
| Thailand I (n=5100) | 5.7 | Malaysia (n=1056) | 30.9 |
| Indonesia II (n=158292) | 5.3 | Indonesia II (n=158292) | 27.9 |
| Indonesia (n=6134) | 0.6 | Indonesia (n=6134) | x |
