# Supplementary material for: Egalitarian Values and Sexual Behavior—The Role of Country Level Values in Shaping Individual Level Behaviors in Africa, South America and Asia
Source: Int J Sex Health. 2021 May 12;33(3):396–409. doi: 10.1080/19317611.2021.1919952 (PMC10929577; doi:10.1080/19317611.2021.1919952)
Supplement: Supplemental Material [file WIJS_A_1919952_SM7227.docx]

| Table S1. Country level descriptive data on all countries | | | | | | | |  |  |
| --- | --- | --- | --- | --- | --- | --- | --- | --- | --- |
| Country | **N** | **GSHS** | **WVS** | **GDP/ppp** | **GII** | **EVI*** | **BOYS (%)** | **AGE**** | **HUNGER**** |
| Argentina (I) | 3958 | 2007 | 2006 | 16.2 | 37 | 48 | 49 | 15 | 1 |
| Argentina (II) | 56736 | 2012 | 2000 | 20.1 | 37 | 52 | 47.8 | 15 | 1 |
| Bangladesh | 2989 | 2014 | 2018 | 3.9 | 55 | 30 | 40 | 14 | 3 |
| Bolivia | 3696 | 2012 | 2017 | 6.7 | 47 | 39 | 50.7 | 14 | 2 |
| Chile (I) | 16262 | 2004 | 2013 | 10.7 | 41 | 46 | 49.2 | 14 | 1 |
| Chile (II) | 4096 | 2013 | 2012 | 22 | 35 | 51 | 50.1 | 14 | 1 |
| Ghana | 12472 | 2007 | 2007 | 2.5 | 58 | 29 | 52.4 | 15 | 3 |
| Guatemala (I) | 11184 | 2009 | 2004 | 5.9 | 56 | 41 | 45.6 | 14 | 1 |
| Guatemala (II) | 4347 | 2015 | 2019 | 8.1 | 50 | 47 | 49.4 | 14 | 1 |
| Indonesia (I) | 6232 | 2007 | 2006 | 6.8 | 54 | 34 | 47.7 | 14 | 2 |
| Indonesia (II) | 11142 | 2015 | 2015 | 12.1 | 46 | 33 | 45.8 | 14 | 2 |
| Malaysia | 51014 | 2012 | 2012 | 23.1 | 30 | 36 | 50 | 15 | 2 |
| North Macedonia | 4228 | 2007 | 2001 | 7.6 | X | 41 | 49.5 | 15 | 1 |
| Peru | 5764 | 2010 | 2012 | 10.6 | 42 | 44 | 49 | 15 | 2 |
| Thailand (I) | 5534 | 2008 | 2007 | 12.1 | 33 | 37 | 49.4 | 14 | 2 |
| Thailand (II) | 11788 | 2015 | 2013 | 15.8 | 35 | 37 | 43.1 | 14 | 2 |
| Trinidad & Tobago (I) | 5938 | 2007 | 2006 | 28.3 | 35 | 40 | 48.5 | 14 | 1 |
| Trinidad & Tobago (II) | 5622 | 2011 | 2010 | 30.8 | 35 | 39 | 54.6 | 14 | 1 |
| Uganda | 6430 | 2003 | 2001 | 0.9 | 62 | 30 | 51.4 | 15 | 2 |
| Uruguay | 7048 | 2012 | 2011 | 18.5 | 32 | 54 | 46.5 | 14 | 1 |
| Venezuela | 8830 | 2003 | 2000 | 10.9 | 48 | 43 | 44.7 | 13 | 1 |
| Zambia | 4514 | 2004 | 2007 | 2.4 | 60 | 40 | 48.9 | 15 | 3 |
| Zimbabwe | 11330 | 2003 | 2001 | 2.2 | 61 | 30 | 44.6 | 15 | X |

*Emancipative Values Index. 100 = strong values. 0 = weak values
**Median since category variable (1–5)
GDP/ppp have been divided by 1000. It should be read as GDP per capita. ppp ($1000)
GII 100 = Equality. GII 0 = inequality
